# Prevention care for secondary health conditions among people living with spinal cord injuries: research protocol

**DOI:** 10.1186/s13104-019-4202-7

**Published:** 2019-03-28

**Authors:** Sonti Pilusa, Hellen Myezwa, Joanne Potterton

**Affiliations:** Department of Physiotherapy, School of Therapeutic Sciences, Faculty of Health Sciences, University of the Witwatersrand, 7 York Road, Johannesburg, 2193 South Africa

**Keywords:** Injuries, Spinal cord, Spinal cord trauma, Preventative care, Prevention intervention, Secondary complications, Secondary conditions

## Abstract

**Objective:**

People living with spinal cord injuries are at a high risk to experience preventable secondary health conditions in their lifetime, which can lead to rehospitalisation and death. Given the fact that spinal cord injury is a long term disability requiring on-going care, there is need to strengthen prevention of secondary health conditions. This study aims to establish factors influencing prevention care for secondary health conditions among people living with spinal cord injuries in a metropolitan area in order to develop a prevention model of care.

**Results:**

A record review of patients living with spinal cord injuries will be conducted to identify the prevalence of secondary health conditions and associated factors. Semi-structured interviews will be conducted on patients living with spinal cord injuries, their caregivers and therapists to explore the contextual factors (personal and environmental factors) influencing the prevention of secondary health conditions. Thematic analysis will be used to identify the themes. Nominal group technique will be used to develop the prevention model of care for secondary health conditions. This study will be conducted at a tertiary and specialised rehabilitation hospital in South Africa.

## Introduction

Globally the incidence of spinal cord injury (SCI) has been reported to range between 40 to 80 cases per million, the prevalence being higher for traumatic spinal cord injuries compared with the non-traumatic SCI [1]. In developing countries the incidence of spinal cord injuries ranges from 2.1 to 130.7/million/year, the majority being young males [2]. To date, there is no national prevalence data for spinal cord injuries in South Africa. However, the current available data indicates a high incidence of spinal cord injuries in Cape Town at 75.6 people per million, mainly due to violence (assault and motor vehicle accidents) [3]. The lack of epidemiological data is troubling considering that the main causes of spinal cord injuries (namely violence, HIV and TB) are in the top ten causes of mortality and morbidity in South Africa [4, 5]. Given that there is no cure for spinal cord injury, an understanding of the health status, long term care needs and secondary complications is essential to inform prevention care practice.

People living with spinal cord injuries (PLSCI) develop preventable secondary complications (secondary health conditions) throughout the journey of disability, that worsen the primary disability and can lead to re-hospitalization and death if not managed properly. Secondary health conditions (SHCs) are conditions that develop over time as a result of the primary disability but are not caused by the primary disability, for example, pressure ulcers, urinary tract infection, spasms and pain [6]. High prevalence of these complications among people with spinal cord injuries has been reported [7, 8]. A scoping review on the frequency of secondary health conditions in individuals with spinal cord injury found that the most reported conditions with a high prevalence rate (50% and higher) were pain, bowel and bladder regulation problems; muscle spasm; fatigue; heart burn and osteoporosis [7]. A recent study by Mashola et al. [9]. looked at readmission rates and prevalence of secondary health conditions in people with spinal cord injury from 2008 to 2012 in a private spinal hospital based in South Africa. The study found the prevalence of pressure ulcers ranged from 39 to 60% with each readmission and tended to get higher over time. The trend was similar for urinary tract infections although the prevalence was not as high. This study recommended further research on prevention protocols to reduce readmission rates for secondary health conditions. Previous research confirms that people with spinal cord injuries continue to struggle with the challenges posed by preventable secondary health conditions and these challenges are exacerbated post discharge [8].

Persons living with spinal cord injuries need health promotion and prevention services to enhance their wellbeing and quality of life [10, 11]. This means a person living with a spinal cord injury must know how to self-manage, prevent the occurrence SHC and be able to manage SHC if they do occur. On the contrary, evidence indicates that long-term health care in terms of health promotion and prevention services for people with disabilities including spinal cord injuries has not been given enough attention either globally or in South Africa [11–13]. A study by Van Loo et al. [12] described care received, care needs and preventability of secondary conditions according to persons with long-term spinal cord injury (SCI) living at home in the Netherlands. Forty seven percent of the participants received the needed care but 41% still needed extra care. This study indicates that prevention care needs of PLSCI were not fully met. There is a need to understand the experiences of secondary health conditions experienced by people living with spinal cord injuries, how these secondary complications are prevented and factors influencing prevention care for these complications. Thus, the aim of this study, over five phases is to establish factors influencing prevention care for secondary health conditions among people living with spinal cord injuries and to propose a prevention model of care for secondary health conditions.

This manuscript will outline the research proposal used to meet the aim of the study.

Table 1 gives a summary of the methodology for the study across the five phases outlining the study objectives; data needed; study design; location; sources of data; sample size; procedure and data analysis.

**Table 1:** Summary of the study

	Phase 1	Phase 2	Phase 3	Phase 4	Phase 5
Objectives	To identify the prevalence of secondary health conditions (SHC) and associated factors	To identify the range of prevention strategies for secondary health conditions experienced by people living with spinal cord injuries (PLSCI)	To explore experiences of SHC and strategies used to prevent SHC	To explore contextual factors influencing prevention of SHC among PLSCI	To develop a prevention model of care for SHC
Data needed	SHC and associated factors (types, frequency)	Prevention strategies for SHC	Experiences of SHC and prevention strategies	Personal factors Environmental factors influencing prevention	Components for the model of care
Study design	Record review	Scoping review	Semi-structured interviews	Semi-structured interviews	Nominal group discussion
Location	Tertiary hospital and rehabilitation hospitals		Rehabilitation hospital	Rehabilitation hospital	Rehabilitation hospital
Source of data	Patient records	Relevant literature (published and unpublished) All study designs	People living with spinal cord injuries (PLSCI)	PLSCI, caregivers Health professionals (occupational therapists, physiotherapists, dieticians, speech therapist, social worker, psychologist)	Group 1: Care users—PLSCI and caregivers Group 2: Experts in SCI rehabilitation therapists, lecturers Group 3: Rehabilitation managers and policy makers
Sample size	323 patients records		Minimum 15 people with spinal cord injuries	Minimum 15 people with spinal cord injuries Minimum 15 caregivers Minimum 15 health professionals	5 to 9 participants per group
Inclusion criteria	PLSCI records admitted at a tertiary hospital (Jan 2016–September 2017) PLSCI using OPD clinic at a rehabilitation hospital (Jan 2016–September 2017)	Population: People living with spinal cord injuries female and male, 18 years and above Concept: Prevention strategies for secondary health conditions in terms of rehabilitation, health promotion, assistive technology and policy Context: community, home setting and clinical based studies (hospital and primary health care centres)	PLSCI attending OPD clinic	PLSCI at the OPD clinic Caregivers of PLSCI Health professionals at the rehabilitation hospital	PLSCI and caregivers from phase three Therapists, Lecturers who teach spinal cord injury; Rehabilitation managers; Policy makers
Procedure and instrumentation	Questionnaire	Joanna Briggs Institute Reviewers Manual 2015: Methodology for JBI scoping reviews	Interviews Interview guide	Interviews Interview guide	Nominal group technique
Data analysis	Descriptive statistics Frequencies and percentages Logistic regression analysis	Narrative summary on the number of studies found addressing the study question, study designs, the range of prevention strategies for secondary health conditions	Thematic content analysis	Thematic content analysis	Qualitative data: thematic content analysis Quantitative data: descriptive statistics

## Main text

This study is designed as a mixed methods study with five phases. Phase one is a cross-sectional study using a record review to identify the prevalence of secondary health conditions and associated factors (type and level of lesion, duration of the disability, age, risky behaviour) in people living with spinal cord injuries. Phase two is a scoping review of evidence to identify the range of prevention strategies for secondary health conditions (protocol submitted to the JBI database). Phase three and four are qualitative studies that seek to explore participant’s experiences of secondary health conditions as well as prevention strategies used by people living with spinal cord injuries and the contextual factors (personal and environmental factors) influencing prevention of secondary health conditions in this population This information will be collected through individual semi-structured interviews. Phase five will involve the development of a prevention model of care for secondary health conditions for people living with spinal cord injuries using the nominal group technique.

### Subject selection

In phase one, the hospital records of patients with spinal cord injuries from January 2016 to September 2017 will be reviewed. The hospital records will be from a tertiary hospital and a rehabilitation hospital, based in Gauteng province, South Africa. For phase three and phase four, participants will be recruited at a rehabilitation hospital and will include patients with spinal cord injuries, their caregivers and allied health professionals. For phase five, a nominal group discussion will be conducted with three focus groups i.e. care users (people living with spinal cord injuries and their caregivers), clinical experts in spinal cord injury rehabilitation therapists and lecturers, and rehabilitation hospital managers and provincial rehabilitation services policy makers.

This study has been approved by the Human Research Ethics Committee of the University of the Witwatersrand (M170938), University of Pretoria Research Ethic Committee (36/2018) and registered with the South African National Health Research Database (reference GP201712036). Permission has been granted by the hospitals. Written, informed consent will be obtained from all the participants.

### Sample size and inclusion/exclusion criteria

For the first phase, sample size was calculated using data from a previous study that looked at the prevalence of secondary health conditions in people living with spinal cord injuries [14]. The Naing et al. [15] sample size calculator for prevalence studies was used to calculate sample size using a power of 95% and a level of significance of 5%, and a minimum of 323 records will be reviewed. All hospital patient records for people living with spinal cord injuries who were admitted to the hospital during the period January 2016 to September 2017 will be reviewed. Hospital patient records for people living with spinal cord injuries who attended the outpatient clinic at the hospital during the period January 2016 to September 2017 will also be reviewed.

For phase three and four, a minimum of 15 individual semi-structured interviews will be conducted for people with spinal cord injuries; caregivers and the allied health professional working with patients with spinal cord injuries respectively. Data will be collected till saturation is reached. For phase five, nominal group discussion will be conducted with three groups, each group will have five to nine participants. Group one will comprise of therapists and lecturers; group two will comprise of people living with spinal cord injuries and their caregivers and group three will be the rehabilitation managers and rehabilitation service policy makers.

### Study procedures

In phase one, each patient record will be retrieved and data will be extracted using a questionnaire sheet to collect information on the demographic data, type of secondary health conditions, spinal cord injury information (duration since injury, level and cause of injury) and associated risk factors. In phase three the principal investigator will interview the participants with spinal cord injuries to explore experiences of secondary health conditions and strategies used to prevent secondary health conditions and factors influencing prevention care. In phase four the caregivers and the health therapist will be interviewed to explore factors influencing prevention of secondary health conditions among people living with spinal cord injuries. The interviews will be audio-recorded and transcribed verbatim. Data gathered in phases one to four will presented to the participants recruited for phase five and nominal group discussion will be conducted as outlined by Cantrill et al. [16] to identify key components of a prevention model of care for secondary health conditions.

### Data analysis

Data from the record review will be analysed using the Statistical Package for the Social Sciences (SPSS) version 22. Significance will be set at p value ≤ 0.05. Regression analysis will be done to assess the associations between the secondary conditions and associated factors (type and level of the lesion; duration of the disability; age; presence of chronic diseases and risky behaviour). Thematic content analysis will be utilised to identify themes from the qualitative data: Data will be coded, similar foci will be categorised and sub-categorised, and thereafter themes will be identified. Coding will be done by the principal investigator and a second coder in two separate sessions and the results will be compared. To ensure trustworthiness of data, an audit trail throughout the research process will be kept in a field journal; detailed description of the data collection, analysis and interpretation will be outlined. Debriefing sessions will take place to discuss the research process, findings and data analysis with the research supervisors [17]. MAXQDA software will be used to analyse and manage the data. Thematic content analysis and descriptive statistics will be used to analyse data from phase five.

## Conclusion

Sustainable Development Goal number three; “to ensure healthy lives and promote wellbeing for all” includes people with disabilities [18]. One way of promoting health for people living with spinal cord injuries is through the prevention of secondary complications. The insight gained from this study will contribute to the body of knowledge on secondary complications experienced by people living with spinal cord injuries, shed light on prevention strategies and the factors influencing prevention care. Furthermore the clinicians working with people with spinal cord injuries can use this data for screening purposes and to develop prevention strategies for commonly experienced secondary health conditions. Lastly, policy makers will be able to utilise the findings of this study to inform health promoting policies and to improve the model of care for persons living with spinal cord injuries.

## Limitations

The study site will mostly be based in one rehabilitation hospital, thus the findings may not be easily generalised to different contexts. Using purposive sampling may increase responder bias. Nevertheless, this study will give us an understanding of the contextual factors influencing prevention and a prevention model of care informed by the different stakeholders i.e. people with spinal cord injuries, caregivers, rehabilitation therapists and managers, academics and policy makers.

## Abbreviations

SCI: spinal cord injury
PLSCI: people living with spinal cord injuries
SHC: secondary health conditions
HIV: Human Immunodeficiency Virus
OPD: outpatient department
